# Supplementary material for: Is Google Trends a useful tool for tracking mental and social distress during a public health emergency? A time–series analysis
Source: J Affect Disord. 2021 Nov 1;294:737–44. doi: 10.1016/j.jad.2021.06.086 (PMC8411666; doi:10.1016/j.jad.2021.06.086)
Supplement: Supplementary file 1 [file mmc1.docx]

Supplementary Figure 1 – Number of COVID-19 cases and deaths in the UK between 21/03/2020 and 21/08/2020*


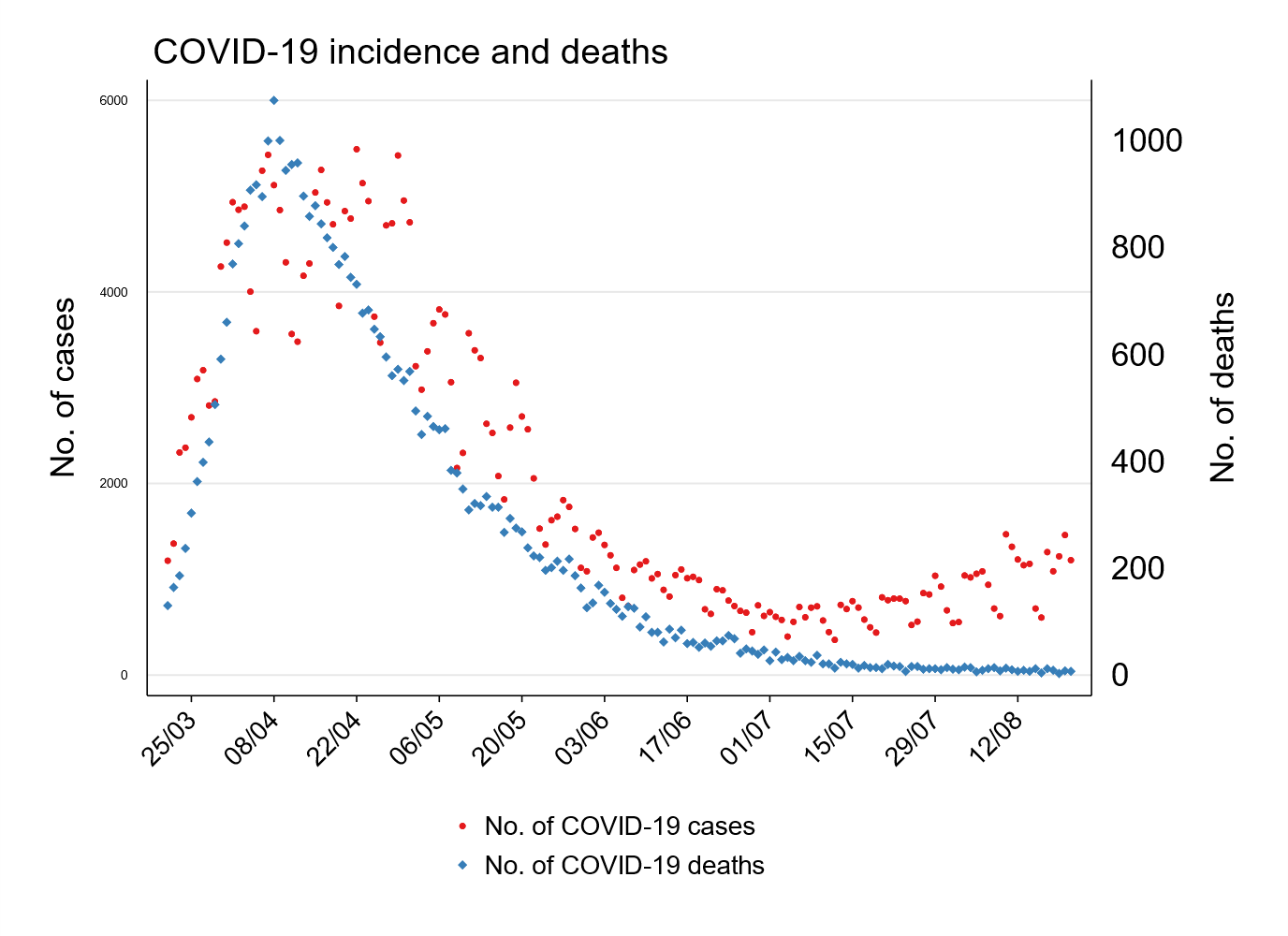


*Data downloaded from <https://coronavirus.data.gov.uk/details/about-data> (21/05/2020)

Supplementary Table 1 – Granger causality test results for the association between weighted self-reported and Google searching time trends data.

|  |  |  | Granger causality test p-values | | | |
| --- | --- | --- | --- | --- | --- | --- |
|  |  |  | Self-reported data precedes Google searching | | Google searching precedes self-reported data | |
| **Mental distress** | | |  | |  | |
|  | Depression | | <0.001 | | - | |
|  | Anxiety | | 0.36 | | - | |
|  | Suicide ideation^b^ | | |  | |  |
|  |  | Self-harm/suicidal ideation | 0.08 | | - | |
|  | Self harm^b^ | |  | |  | |
|  |  | Self-harm | 0.43 | | 0.43 | |
|  | Suicide^b^ | |  | |  | |
|  |  | Self-harm | 0.62 | | 0.99 | |
|  |  | Self-harm/Suicidal ideation | 0.04 | | - | |
| **Social distress** | | |  | |  | |
|  | Loneliness | | <0.001 | | - | |
|  | Abuse | | 0.01 | | - | |

^a^VAR – Vector autoregressive. The number of lags is the number of days between one time trend (e.g. self-reported) and the other (e.g. Google searches).

^b^Google search topics were compared to self-reported self-harm and self-harm/suicidal ideation time trends data
